# Neuroprotective cellular and molecular mechanisms of physical exercise on neurodegenerative diseases

**DOI:** 10.5599/admet.3058

**Published:** 2026-03-11

**Authors:** Saswati Swagatika Sahoo, Pratap Kumar Sahu, Nishigandha Sa, Anindita Behera

**Affiliations:** 1 School of Pharmaceutical Sciences, Siksha ‘O’ Anusandhan Deemed to be University, Bhubaneswar, Odisha, India; 2 Amity Institute of Pharmacy, Amity University, Kolkata, West Bengal, India

**Keywords:** Physical exercise, neuroprotective, neurogenesis, neuronal survival, neuroendocrine regulation, neurotrophins

## Abstract

**Background:**

Neurodegenerative diseases such as Alzheimer's, Parkinson's, and Huntington's disease are characterized by a progressive loss of neuronal function and loss of synaptic capacity. Physical exercise (PE) is one of the non-clinical techniques for the management of brain health and neurodegeneration.

**Mechanisms:**

PE enhances the body's metabolic functions through cellular and molecular changes. It trades off metabolic functions, energy expenditure, and signalling processes to ensure physiological homeostasis and defence against disease. Exercise produces cascades, at the molecular level, including neurotrophic signalling, similar to those generated by drugs. It increases the levels of the brain-derived neurotrophic factor (BDNF), insulin-like growth factor 1 (IGF-1), and vascular endothelial growth factor (VEGF). These elements favour the growth of new neurons, vascular enlargement, and synaptic plasticity. PE also induces microglial cells to attain a neuroprotective, anti-inflammatory phenotype, reduces detrimental cytokines, promotes cellular clearance through autophagy, restores neurotransmitter homogenisation, and induces hippocampal cell formation. Collectively, it acts as a powerful modulator of health and brain activity.

**Implications:**

The aggregate processes enhance neuronal vulnerability to harm, aid cognitive functioning, and ensure the stability of neural networks.

**Conclusion:**

PE is an exciting additive therapy for preventing and treating various neurodegenerative disorders by orchestrating a diverse array of cellular and molecular responses.

## Introduction

Physical exercise (PE) has been recognized as an effective non-pharmacological treatment that has far-reaching therapeutic implications on the physical (corporeal) and the neural systems [1]. Biologically, PE can be defined as a specialised type of physical activity, which is premeditated, designed and repetitive and is performed primarily to improve and maintain physical fitness and general well-being. [2]. The World Health Organization (WHO) reliably emphasizes that regular PE is a cost-effective and accessible means of preventing chronic diseases, which act as a keystone for a healthy life. Along with the effects on musculoskeletal, cardiovascular, and metabolic health improvement, exercise has increasingly been recognised as a systemic regulator of health, influencing endocrine balance, immune function and brain physiology [3].

Patients incur a significant health burden in the world in case of neurodegenerative diseases like Alzheimer's disease, Parkinson's disease (PD), epilepsy and Huntington's disease (HD). The conditions result in the gradual deterioration of the cognitive functions, motor impairment and worsening of the quality of life [4,5]. They are primarily defined by the loss of neurons, impairment of synapses, mitochondrial injury, aggregation of proteins and chronic neuroinflammation, which collectively disrupt brain homeostasis [6]. Existing pharmacological treatments provide symptomatic relief and, in most cases, are not effective at preventing disease progression [7]. Thus, it has become more urgent to identify non-pharmacological treatment methods that can regulate these harmful mechanisms and keep the brain healthy. Physical exercise (PE) is an inexpensive, readily available, and powerful neuroprotective intervention that has attracted considerable interest [8]. Various research findings indicate that aerobic and resistance training can reduce cognitive impairment, lower the risk of dementia, and slow the progression of neurodegenerative diseases [9].

The last twenty years have provided strong evidence from literature reviews, pre-clinical studies, and randomized clinical trials that regular PE has neuroprotective effects [10,11]. In addition to slowing the disease, this type of exercise maintains cognitive and neuro-resilience across a variety of cellular and molecular processes. PE can improve neurotrophic signalling at the cellular and molecular level by upregulating brain-derived neurotrophic factor, vascular endothelial growth factor (VEGF) and insulin-like growth factor-1 (IGF -1) [12]. All these together favour neurovascular integrity, neurogenesis, and synaptic plasticity, thus favouring memory and learning. In addition, PE also enhances mitochondrial activity and antioxidant adaptations through the PGC1 alpha pathway, thus reestablishing redox balance and relieving oxidative stress [13]. Besides these, PE reduces neuroinflammation through suppression of pro-inflammatory cytokines and creation of an anti-inflammatory environment [14,15]. In addition to having direct effects on the nervous system, PE promotes vascular health, including angiogenesis, improved endothelial activity, and maintenance of the blood-brain barrier (BBB) integrity [11,16]. Such changes in vascularity help supply metabolic substrates and improve the clearance of neurotoxic by-products, which are vital for sustaining neurons. In addition, exercise-induced neurogenesis and synaptic reorganisation enhance cognitive resilience and prevent neurodegeneration [17]. These benefits extend systemically through the "exercise responsome," a coordinated response involving communication among multiple organs. Various signalling molecules such as myokines, adipokines, hepatokines, and osteokines, collectively known as exerkines, regulate metabolism and even influence brain function [18]. Notably, some exerkines can cross the BBB and activate protective signals in the brain, including IGF 1/PI3K/AKT or AMPK/SIRT1/PGC 1 1 axis, to induce cellular survival and neuroprotection [19,20].

The overall effects of these processes imply that PE provides a multifactorial neuroprotective cover and works through the convergence of various mechanisms that maintain cerebral health. Unlike pharmacological treatments that usually focus on a single molecular pathway, PE leads to system-wide effects by simultaneously rhythmizing neurotrophic signalling, mitochondrial dynamics, oxidative stress response, inflammatory processes, vascular health and synaptic connections. All these pleiotropic effects make exercise a top-ranking lifestyle intervention for delaying and reducing, or even avoiding, the development of neurodegenerative diseases. The goal of the review is to evaluate the current evidence about the neuroprotective cellular and molecular processes triggered by PE, and more specifically, in reference to the applicability of these processes in relation to neurodegenerative diseases. To ensure methodological transparency and rigour, a full search of the PubMed, Scopus, and Web of Science databases was performed, selecting literature published between the years 2015 and 2025.

## Physiological responses to physical exercise

PE acts as a multisystemic therapeutic agent by coordinating responses across the skeletal muscles, adipose tissue, and the CNS [3]. In the skeletal muscle, it acts as a potent remodelling ligand, driving a fibre-type switch toward oxidative phenotypes with increased mitochondrial biogenesis, vascularisation, glucose uptake, and insulin sensitivity, thereby improving systemic metabolic efficacy. In adipose tissue, exercise acts as a metabolic adjuvant, converting white adipose tissue into a more oxidative, mitochondria-rich, and endocrine-active state, while brown adipose tissue (BAT) remains a thermogenic catalyst with less defined contributions [21]. Beyond peripheral metabolism, PE exerts neuroactive pharmacodynamics, enhancing hippocampal neurogenesis, synaptic plasticity, and long-term potentiation while upregulating a repertoire of growth factors (BDNF, IGF-1, VEGF), neurotransmitters (GABA, dopamine, glutamate, serotonin), and transcriptional regulators (Sox2, FoxOs, NeuroD, Pax6, Neurog2, Klf9) [22]. These molecular and cellular mediators act as endogenous co-therapeutics, conferring both neuroprotection and cognitive enhancement, with human studies showing increases in brain volume after aerobic exercise in older adults. Understanding the variables of exercise “dosing” (intensity, duration, modality) alongside the development of exercise mimetics holds promise for replicating these systemic and CNS-targeted actions pharmacologically [23].

### Physical exercise and the brain

**Figure 1.**
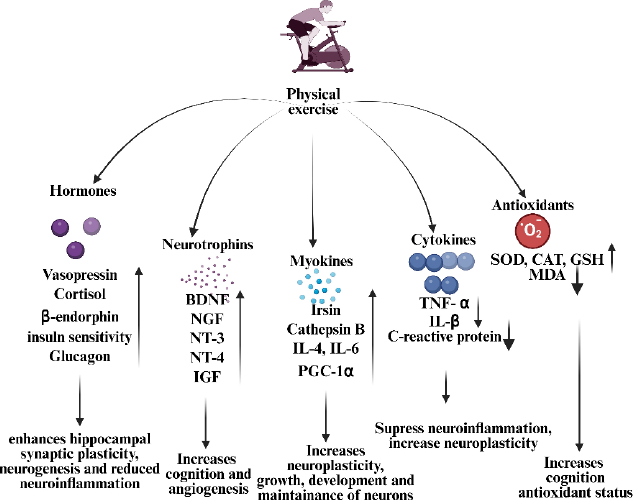
Schematic representation of the systemic and neural mediators activated by physical exercise (PE). PE increases the release of hormones and antioxidants, contributing to neuroprotection and improved redox balance. It upregulates neurotrophins and myokines that support cognition, angiogenesis, and neuronal growth. Additionally, exercise modulates cytokine profiles, thereby reducing neuroinflammation and promoting overall brain health

PE is a neuropharmacological modulator with a broad-spectrum effect on the health of the brain and on cognitive performance [24]. Exercise is an endogenous drug delivery system that increases neurotrophins, such as IGF, nerve growth factor (NGF) and BDNF [25]. These molecules are neurotrophic agonists that promote neurogenesis, synaptic receptor plasticity, neuronal survival pathways, and finally protect cognitive pharmacodynamics (Figure 1). Simultaneously, exercise improves cerebral perfusion (dose-dependent augmentation of blood flow), neuroinflammatory signalling cascades, and fortifies synaptic networks of communication [26]. Exercise is neuroprotective and is able to reduce the effects of an ageing brain by improving the structural integrity of the hippocampus and receptor responsiveness [27].

Further, exercise leads to the secretion of endorphins, which are mood-stabilising neuromodulators that decrease stress levels and increase affective tone. Both preclinical and clinical studies indicate that PE elevates grey and white matter volumes, axonal connectivity, and dose-responsive improvements in cognitive functions [24]. For example, Lu et al. studied the effects of treadmill exercise in STZ-induced AD rat models. Treadmill exercise significantly reduced neuronal apoptosis in the hippocampus region and improved cognitive functions [28]. A meta-analysis of 29 randomised controlled trials (n = 2049) found that aerobic exercise improved executive functions, memory, attention, and processing speed [29].

Functional neuroimaging reveals that even acute moderate-intensity exercise enhances dorsolateral prefrontal cortex activation, thereby improving executive function as measured by the Stroop cognitive test. In addition, prolonged training interventions, such as eight weeks of taekwondo practice, have been associated with persistent improvement in cognitive performance, as verified through Stroop task assessments [30]. Other well-established cognitive paradigms, such as the Go/No-Go and Flanker tasks, have similarly been utilized to assess the nootropic potency of exercise.

### Physical exercise and neurogenerative disease

**Figure 2.**
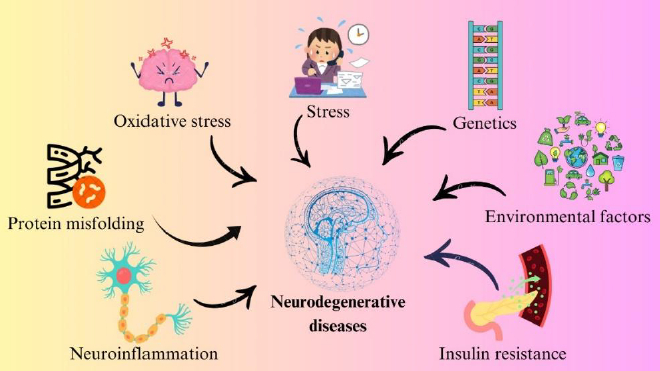
Schematic representation of the major factors contributing to neurodegenerative disorder; like oxidative stress, protein misfolding, neuroinflammation, stress, genetics, environmental factors, and insulin resistance, that interact and collectively drive neuronal damage and degeneration. These interconnected pathways highlight the multifactorial nature of neurodegenerative disorders

The exact causes of neurodegenerative disorders are not fully understood, but they often involve a combination of different phenomena. The pathogenesis of neurodegeneration can be summarised as the influence of multiple factors, including genetic alterations and mutations, environmental factors, insulin resistance in the brain, oxidative stress, neuroinflammation, protein aggregation, and stress [31]. Figure 2 shows a schematic of the factors responsible for neurodegeneration. The following section discusses each factor of neurodegeneration.

The most prevalent type of dementia is AD, a progressive neurodegenerative disorder affecting around 50 million people worldwide, with cases expected to triple by 2050 [32]. Currently, there is no cure for AD, and treatment mainly involves medications and supportive therapies to slow disease progression. Several hypotheses, including decreased cholinergic activity, accumulation of Amyloid-β (Aβ) plaques, and hyperphosphorylated tau, etc., are proposed to explain the pathophysiology of AD [33]. PE is regarded as an effective non-pharmacologic strategy to prevent cognitive impairment, reduce the risk of AD, and improve brain health by enhancing cognitive function, reducing neuropsychiatric symptoms, and providing anti-inflammatory and neuroprotective effects [34, 35]. Pre-clinical studies have shown that PE prevents obesity-induced brain damage by reducing inflammation and enhancing vascular function [36] (Table 1).

**Table 1.** Neuroprotective effects of physical exercise in experimental models of different neurodegenerative disorders

Neurodegenerative disorders	Experimental model	Exercise intervention	Primary molecular target	Alteration by physical exercise	Ref.
Alzheimer’s disease	STZ-induced AD rat model	Treadmill exercise for 13 weeks	Amyloid-β and tau protein	Decrease tau and amyloid beta disposition by upregulating the expression of tripartite motif-containing 9 (TRIM9)	[91]
	2xTg-AD mice	Resistance exercise (5 sessions per week for 7 weeks)	Amyloid-β	Improved memory, mood, and cytokine expression in AD animals, also reduced amyloid beta disposition	[92]
	APP/PS1 mice	Treadmill exercise (8 weeks, 4 sessions/week)	REV-ERBα protein and TFEB pathways	Reduced cognitive deficits, neuroinflammation, and improved mitochondrial biogenesis in the hippocampal region of the mice	[93]
	Transgenic AD mice	Treadmill exercise for 12 weeks	Gut microbiota	Increased gut microbial diversity and the development of dominant strains of probiotics	[94]
	Transgenic old male mice and double transgenic AD mice	Treadmill exercise for 12 weeks	Micro RNA-3473e, NMDA/AMPA receptor signalling	Improved cognitive function and synaptic plasticity by suppressing miR-3473e and upregulating EphB2-NMDA/ AMPA receptor signalling pathway in the early stage of AD	[95]
	Wild-type transgenic AD mice	Treadmill exercise (Twice a day for 1 month)	Amyloid-β peptide	Improved meningeal lymphatic vessel plasticity, mitigated Ad-like pathophysiology.	[96]
	APP/PS1 and C57BL/6 AD mice	Treadmill exercise for 20 weeks	Gut microbiota	Improved cognitive function by altering the gut microbiome in mice	[97]
Parkinson’s disease	6-OHDA-induced PD rat model	Treadmill exercise (3 times a week for 40 minutes)	Cannabinoid receptor type 1 (CB1) and type 2 (CB2), and μ-opioid receptor (MOR)	Promote the antinociceptive effect in PD rats	[98]
	MPTP-induced PD mice model	Aerobic exercise	dopaminergic pathway; AMPK/Sirt1 pathway	Reduced neuronal apoptosis, loss of dopaminergic neurons, and also improved motor function and mitochondrial fission in PD mice	[99]
	Rotenone-induced PD mouse model	Treadmill exercise for 21 days	M1/M2 microglial polarisation pathway and inflammatory cytokine signalling	Reduced motor symptoms, anti-inflammatory cytokines IL-4 and IL-10, and increased pro-inflammatory cytokines TNF-α and IL-β	[100]
	6-OHDA-induced PD rat model	Treadmill exercise for 4 weeks	PGC-1α, NRF-1 and TFAM	Enhanced dopaminergic system in the PD rat brain	[101]
	6-OHDA-induced PD rat model	Treadmill exercise for 16 weeks	AMPK-PGC-1α pathway	Reduced loss of dopaminergic neurons in the PD brain	[102]
	6-OHDA-induced PD rat model	Treadmill exercise for 10 weeks	Brain and muscle lipid remodelling	Improved motor function and restored brain and muscle lipid profile in PD rats	[103]
	MPTP-induced PD mice model	Treadmill exercise	Irisin/AMPK/SIRT1 pathway	Protected dopaminergic neurons in PD mice by decreasing microglia-driven neuroinflammation via the Irisin/AMP/Sirt1 signalling pathway	[104]
	6-OHDA-induced PD rat model	Aerobic exercise (40min/session, 3x per week)	Neurofilament light chain and glial fibrillary acidic protein	Improved motor impairment in PD rats; decreased serum level of biomarkers of PD	[105]
	6-OHDA-induced PD rat model	Treadmill exercise for 15 days	Synaptic vesicle glycoprotein 2A	Protected nigral and striatal synaptic plasticity in PD rats	[106]
Huntington’s disease	Quinolinic acid-induced HD rats	Treadmill exercise (30 minutes once a day for 14 days)	BDNF-TrkB signalling pathway	Improved short-term memory by promoting hippocampal cell proliferation through increasing BDNF expression in HD rats	[107]

Aerobic exercise increases ABCA1 gene expression, which may improve cognitive performance [37,38]. Exercise has been found to stimulate neurotrophic factors, reduce inflammation, promote blood vessel formation, and decrease the aggregation of Aβ plaques and tau protein in the brain [39,40].

The second most prevalent neurodegenerative disease, PD, is characterized by the degeneration of dopaminergic neurons and the accumulation of aberrant α-synuclein protein due to a malfunctioning autophagy-lysosomal system [41,42]. Studies have shown that exercise improves motor dysfunction and cognitive impairment. In general, high-intensity exercise is the most effective mechanism for improving motor symptoms[ ,^44^]. Pre-clinical studies demonstrate that PE preserves dopaminergic function, suppresses α-synuclein pathology, and may delay the advancement of PD, supporting its role as a disease-modifying intervention (Table 1).

HD is a genetic, progressive, and chronic neurodegenerative disease, which involves motor dysfunction, cognitive impairment, and psychiatric disturbances [45]. PE shows a significant impact on the treatment and progression of HD. It helps in improving motor function, balance and mobility. PE provides both physical benefits as well as mental health and cognitive function in HD patients [46]. Research demonstrates that PE alleviates anxiety and depression and also enhances mood. It also delays the progression of motor symptoms and decreases the risk of falls by improving muscle strength, coordination, and cardiovascular fitness [47]. It also helps in preserving brain volume and neuroplasticity in the regions affected by HD, offering neuroprotective benefits.

## Mechanisms involved in neuroprotection

PE plays a central role in preventing and halting the disease progression. The key mechanisms involved in this process include structural and functional mechanisms, pathological mechanisms, molecular mechanisms, neuroimmune and inflammatory mechanisms.

### Structural and functional mechanisms

PE demonstrates neuroprotective effects through various structural and functional modifications in different brain regions and time scales, thereby enhancing neuronal flexibility, which also plays an essential role in preventing neurodegenerative diseases.

#### Synaptic plasticity

The capacity to modify the strength and effectiveness of synaptic transmission from pre-existing neural connections is known as synaptic plasticity, and it is an inherent characteristic of neurons [13]. Most important for memory, learning, and the development of the brain's reaction to injury, synaptic plasticity shifts synaptic strength from milliseconds to hours or days [48]. Synaptic plasticity leads to long-term potentiation (LTP), which increases synaptic strength, and to long-term depression (LTD), a continuous decrease in synaptic strength [49, 50]. PE increases structural plasticity and synaptic proteins such as synapsin I and postsynaptic protein 95 [51]. Pre-clinical research suggests that regular PE improves brain health by improving learning and memory function and countering cognitive decline with ageing [52]. In a pre-clinical study on 4-month-old mice, a 12-week aerobic exercise training program significantly increased synaptic plasticity and improved hippocampal ultrastructure, including the preservation of mitochondria, neurofilaments, and microtubules [53]. PE enhances dendritic remodelling in the hippocampus and cortical neurons by increasing dendritic length, complexity, and spine density [49,54]. These structural and functional modifications provide enhanced cognitive function. Tsai et al. reported that trade mill exercise has a significant neuroprotective effect, reducing dendritic length and decreasing the number of spines in CA1 neurons [55]. It also increases hippocampal synaptic plasticity and preserves dendritic complexity.

#### Hippocampal neurogenesis

PE enhances the hippocampal neurogenesis, or the production of neurons in the dentate gyrus of the hippocampus [56]. It has been demonstrated through animal research that exercise enhances the growth, survival, and maturation of neuronal cells that shield the activities of the hippocampus [57]. Neurogenesis linked to exercise is dose-dependent and confers beneficial effects through neurotrophic factor signalling and increased blood flow to the brain, as well as decreased neuroinflammation [58]. Increased hippocampal neurogenesis is a compensatory mechanism for neuronal loss, and thus, cognitive deficiency is minimised.

#### Cerebrovascular adaptation

Regular PE involves angiogenesis (formation of new blood vessels) in the hippocampus and cortex of the brain [59,60]. It improves the supply of oxygen and nutrients to the brain, the elimination of metabolic waste products, and the vascular density. PE enhances BBB permeability by reducing the infiltration of inflammatory mediators and neurotoxic substances. PE increases the permeability of the BBB by slowing the process of infiltration of inflammatory mediators and neurotoxic substances. It protects against neurodegeneration and cognitive impairment by improving cerebrovascular activity via the generation of endothelial nitric oxide, improving cerebral blood flow, and cerebrovascular responsiveness. Exercise has cerebrovascular benefits in ageing because vascular dysfunction becomes more frequent, leading to neurodegeneration [61].

#### Network connectivity changes

Modern neuroimaging evidence demonstrates that PE enhances the large-scale connectivity of brain networks and enhances communication in sensorimotor and cognitive control functions. PE particularly influences the default mode network (DMN) that is functionally connected in neurodegenerative diseases [62]. Aerobic exercise partially restores DMN connectivity in older adults, suggesting a reversal of pathological alterations through activity-dependent synaptic strengthening and enhanced neurovascular coupling [63]. Likewise, exercise enhances the interconnection of the cortical memory system of the hippocampus, which promotes memory performance through augmentation of network integration and LTP-mediated synaptic efficacy. Structurally, PE strengthens white matter integrity by promoting myelin plasticity and axonal density [7]. These changes are supported by exercise-driven VEGF upregulation and angiogenesis, leading to improved cerebral blood flow and metabolic support for engaged neuronal networks. So, all these cellular and molecular mechanisms collectively converge to reinforce brain network connectivity, thereby improving memory and cognitive performance.

### Pathological mechanism

PE prevents the progression of neurodegenerative diseases by affecting their pathological mechanism.

#### Tau pathology and the amyloid-β hypothesis

Hyperphosphorylated tau proteins and amyloid-β (Aβ) plaques are hallmark pathological indicators of AD [64,65]. Regular PE mitigates these processes through multiple mechanisms. PE reduces tau hyperphosphorylation by modulating key enzymes, such as protein phosphatase 2A (PP2A) and glycogen synthase kinase-3β (GSK-3β), and enhances autophagy, thereby facilitating the clearance of abnormal tau aggregates from the brain [66]. Pre-clinical trials have consistently demonstrated that PE reduces hippocampal and cortical A levels, decreases soluble A2 levels, enhances A2 clearance, and reduces A2 production and brain proteostasis [67] (Table 1). PE also enhances glymphatic and CBF functions by increasing cerebral blood flow, mitigating neuroinflammation, stimulating microglial phagocytosis of A2, and reducing toxic inflammatory reactions [57]. Moreover, exercise enhances the activity of A2-degrading enzymes like neprilysin and insulin-degrading enzyme [68,69]. Human studies show that physically active people tend to have lower levels of amyloid and tau proteins, as detected by brain imaging and fluid biomarkers [70-72]. Although direct evidence of exercise reducing AD pathology in humans is limited, findings from animal studies suggest that regular exercise may help slow the progression of AD by targeting its underlying disease mechanisms.

#### α-synuclein aggregation

**Figure 3.**
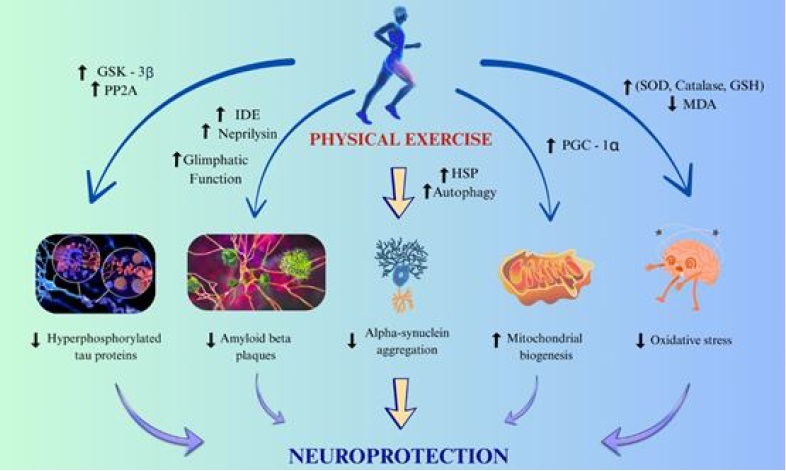
Illustration showing the neuroprotective mechanisms of physical exercise. Exercise enhances enzymatic activity (GSK-3β, PP2A, IDE, and Neprilysin), boosts autophagy and heat shock proteins (HSP), and promotes mitochondrial biogenesis, while reducing oxidative stress and protein aggregation. These processes collectively decrease hyperphosphorylated tau, amyloid-beta plaques, and alpha-synuclein aggregation, thereby promoting neuroprotection

α-synuclein aggregation represents the primary pathological aetiology of PD. Accumulation of α-synuclein disrupts neuronal function and contributes to neurodegeneration, particularly in the dopaminergic region of the substantia nigra in the midbrain [23]. Pre-clinical studies demonstrate that regular PE reduces α-synuclein accumulation and associated neuroinflammation in these brain regions (Figure 3). Leem *et al.* reported that rotarod walking exercise reduced neuroinflammation and α-synuclein oligomerization in a mouse model of MPTP-induced PD [73]. The protective effects of exercise are largely attributed to its capacity to enhance cellular proteostasis mechanisms. Specifically, exercise upregulates protein-folding chaperons such as heat shock proteins (HSPs), activates the autophagy-lysosomal pathway, and strengthens the ubiquitin-proteasome system, thereby facilitating the clearance of misfolded α-synuclein [74,75]. Beyond proteostasis, exercise also protects dopaminergic neurons by decreasing oxidative stress, improving mitochondrial function, and increasing the availability of neurotrophic factors such as GDNF and BDNF [76,77]. Clinical evidence further supports these findings, showing that regular PE improves motor symptoms and enhances dopaminergic signalling in PD patients [78]. Although direct human evidence on α-synuclein pathology remains limited, converging insights from animal studies and clinical benefits strongly support exercise as a promising strategy to slow PD progression.

#### Mitochondrial function

Mitochondrial dysfunction is a primary determinant contributing to the pathogenesis of neurodegenerative diseases, which causes oxidative stress, loss of cellular energy reserves, and eventually leads to neuronal apoptosis [79]. By enhancing the physiological capabilities of mitochondrial organelles, regular PE maintains neuronal energy homeostasis and mitigates this detrimental cascade [80]. A key process involves the activation of peroxisome proliferator-activated receptor-gamma coactivator-1 alpha (PGC-1α) the overall regulator of mitochondrial genesis [81]. This pathway leads to upregulation of mitochondrial enzymes by exercise, increases efficiency of oxidative phosphorylation, and enhances the production of adenosine triphosphate (ATP) [82]. In addition to biogenesis, physical activity has pharmacodynamic-like effects on the overall quality control of mitochondria, inducing mitophagy to eliminate dysfunctional mitochondria and altering mitochondrial dynamics through balanced regulation of fusion and fission processes, thus maintaining mitochondrial integrity [83,84]. In a pre-clinical model, exercise restores hippocampal mitochondrial function and attenuates oxidative stress in AD rat models. It also improves ETC activity and dopaminergic resilience in PD models (Table 1).

#### Excitotoxicity and oxidative stress

Excitotoxicity and oxidative stress represent key pathological pathways underlying many neurodegenerative disorders [85]. PE modulates glutamatergic transmission by regulating N-methyl-D-aspartate (NMDA) receptor activity, enhancing astrocyte-mediated glutamate uptake, and strengthening inhibitory gamma-aminobutyric acid (GABA) signalling, thereby limiting excitotoxic neuronal injury [86]. In addition, regular PE enhances the brain’s antioxidant activity by upregulating enzymes such as superoxide dismutase (SOD), catalase, and glutathione peroxidase, which collectively contribute to the reduction of oxidative stress [87-89]. Regular PE reduces the death of neurons by lowering lipid peroxidation, protein oxidation, and DNA damage in areas of the brain that are highly susceptible to neurodegeneration (Figure 3) [11]. The exercise-induced neuroprotection against excitotoxicity and oxidative stress can be seen in several diseases, such as amyotrophic lateral sclerosis and Huntington's disease [90].

### Molecular mechanisms

#### Neuroendocrine regulation

PE acts as a significant physiological stressor, capable of activating the neuroendocrine system. It stimulates the hypothalamic–pituitary–adrenal (HPA) axis, elevating glucocorticoid levels, which support cognitive function and enhance neuroplasticity [108,109]. Over time, regular exercise improves the regulation of the HPA axis, helping buffer the undesirable effects of chronic stress on the brain, which are known contributors to neurodegenerative diseases. Adrenocorticotropic hormone, vasopressin, cortisol, β-endorphin, and several other hormones are altered by PE from resting levels [110] (Table 2). It functions as a potent stimulant for the HPA axis. The type of exercise (intensity, aerobic, duration, and strength), the time of day, the meal consumed, and the subject's attributes (gender and prior training) all influence the nature of the stimulus.

**Table 2.** Hormone regulation by physical exercise

Hormone regulation	Functions of the hormone	Ref-
Releases hormones from the hypothalamus and controls AMPK and mTOR signalling	Increase coordination between the muscle-brain axis.	[49,122]
Increases insulin sensitivity	Increases glucose uptake by muscles, prevents glycaemic neurotoxicity, and reduces the risk of diabetes-induced cognitive impairment.	[114]
Releases beta-endorphin	Increase neurogenesis and reduce neuroinflammation.	[115]
Release vasopressin	Increase hippocampal synaptic plasticity and prevent Aβ-induced cognitive impairment.	[116]
Release cortisol from the adrenal gland.	It acts as a stress hormone, reducing neuroinflammation and oxidative stress in the brain.	[123]

The hypothalamus, in the brain, acts as a control centre during exercise (Table 2). In response to PE, the hypothalamus exhibits a metabolic response like that of muscle tissue. Hypothalamic tissues show an increase and decrease in AMP-activated protein kinase (AMPK) and mammalian Target of Rapamycin (mTOR) signalling, which increases coordination within the muscle-brain axis and improves the systemic response to PE [111].

The endocrine pancreas secretes insulin and glucagon, both of which are necessary regulators of glycaemia. Exercise has been shown to increase insulin sensitivity, thereby enhancing glucose uptake by skeletal muscle cells [112, 113] (Table 2). A stable glucose level prevents glycaemic neurotoxicity and reduces the risk of diabetes-induced cognitive impairment [114]. Pre-clinical research suggests that both insulin and glucagon decrease CSF glutamate levels in the brain and exhibit neuroprotective effects in diabetic rats.

Beta-endorphins are endogenous opioids, neuropeptides released from the pituitary gland and hypothalamus in response to various stimuli, including exercise [115]. PE increases the level of circulating beta-endorphins in the body. Beta-endorphin acts as a natural analgesic, reducing pain perception, and can have mood-enhancing effects. It also shows neuroprotective effects, including neurogenesis and reduced inflammation (forming new neurons) in certain brain regions [115] (Table 2).

Vasopressin is a neuropeptide hormone released in the hypothalamus that regulates water balance, blood pressure, and stress responses [116]. During exercise, vasopressin secretion may increase to help maintain water and electrolyte balance, especially during strenuous or prolonged PE. Vasopressin enhances hippocampal synaptic plasticity and prevents amyloid-β-induced impairment of LTP [117]. In rat hippocampal slices, vasopressin supports excitatory postsynaptic potentials and promotes LTP in the CA1/subiculum and the dentate gyrus [118]. It exhibits both anticonvulsant and proconvulsant effects, and its metabolites enhance memory by aiding in consolidation and retrieval (Table 2).

Several key stress mediators, including cortisol released during high-intensity exercise, negatively affect hippocampal plasticity by inhibiting neurogenesis due to extreme activation of the hypothalamic-pituitary-adrenal (HPA) axis [119,120]. The cortisol spurt caused by activity, on the other hand, elicits adaptive mechanisms that increase the brain's ability to withstand stress and potentially produce neuroprotective effects [121].

#### Neurotransmitter regulation

PE broadly modulates central monoaminergic networks, particularly the dopaminergic, serotonergic, and noradrenergic systems [124]. Several research studies indicate that PE influences the synthesis and metabolic processing of monoamine neurotransmitters, including dopamine, serotonin, and noradrenaline, thereby affecting their neural activity [125]. PE promotes the release of neurotransmitters and plays a key role in mood regulation, stress reduction, and overall psychological well-being. Through its neurochemical effects, PE supports mental health by modulating neurotransmitter activity. Exercise-induced increases in circulating calcium can influence brain function by activating the enzyme tyrosine hydroxylase, a calcium- and calmodulin-dependent process that contributes to dopamine (DA) synthesis [25]. Exercise decreases the release of norepinephrine, thereby hyperpolarising noradrenergic neurons and reducing their firing frequency [126]. In comparison to sedentary people, PE increases the level of NE in the spinal cord and pons-medulla. It also boosts norepinephrine's endogenous activity. These results suggest a possible connection between norepinephrine and enhanced cognitive function. It regulates 5-HT levels in the brain, enhancing neuroprotection by increasing the synthesis and release of 5-HT, particularly in the hippocampus and prefrontal cortex, thereby regulating mood, cognition and stress. Increased 5-HT during PE promotes neurogenesis and synaptic plasticity, reduces inflammation and oxidative stress, and protects against neurodegeneration [127,128]. Consequently, exercise enhances brain well-being, elevates mood, and helps prevent disorders such as AD and PD [129]. Nonetheless, a major change in hippocampal 5-HT level occurred after seven days of intensive treadmill exercise.

#### Neurotrophins and neurotropic factor

**Figure 4.**
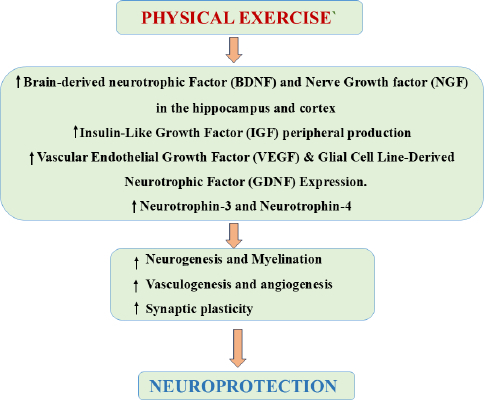
Schematic representation of role of Neurotrophins in Physical Exercise-Induced Neuroprotection through cellular and molecular pathways. PE increases key neurotrophic factors, like BDNF, NGF, IGF, VEGF, and NT-3 and NT-4, promoting neurogenesis, myelination, angiogenesis, and synaptic plasticity. These combined effects strengthen brain resilience and contribute to overall neuroprotective outcomes

The neurotrophins are the primary mediators of the positive effects of PE in the brain (Figure 4). Neurotrophins are essential for maintaining neurogenesis, cognitive, structural, and functional brain plasticity [130]. These growth factors control neuronal growth, differentiation, and survival and axon, dendrite, and synaptic plasticity development in diverse regions of the brain. Regular PE also induces the production of neurotrophins that protect cognitive functioning, promote neurogenesis, and fight age-related cognitive impairments [131,132]. PE regulates important neurotrophic factors like NGF, BDNF, VEGF, GDNF, IGF, neurotrophin-3 (NT-3) and neurotrophin-4 (NT-4) (Table 3) [133].

**Table 3.** Summary of major clinical trials investigating the neuroprotective effects of physical exercise in different neurodegenerative diseases

Trial name/ID	Sponsor	Disease	Exercise type/duration	Study duration	No. of population	Current stage
NCT02384993	University of Wisconsin, Madison	Alzheimer’s disease	26 weeks of Aerobic exercise	2015-04-28 to 2016-07-19	24	Completed
NCT01128361	Jeff Burns, MD	Alzheimer’s disease	150 minutes a week of aerobic exercise over 3-5days.	2010-05 to 2015-04	76	Completed
NCT01129115	Jeff Burns, MD	Alzheimer’s disease	Aerobic exercise	2010-05 to 2014-04	101	Early phase 1
NCT00403507	Intermountain Health Care, Inc.	Alzheimer’s disease; Memory disorder	Aerobic exercise programme and strength training.	2006-10 to 2009-10	79	Phase 2
NCT05597124	Rutgers, The State University of New Jersey	Alzheimer’s disease	aerobic cardio-dance fitness exercise	2023-04-20 to 2027-08-31	280	Phase 2
NCT01681602	Rigshospitalet, Denmark	Alzheimer’s disease	12 weeks of aerobic exercise	2012-01 to 2014-06	200	Phase 3
NCT01183806	Federal University of Bahia	Alzheimer’s disease	Exercise training program	2010-07 to 2012-03	40	Phase 3
NCT03696589	Teachers College, Columbia University	Parkinson’s disease	Physical Activity	2018-05-21 to 2019-04-12	13	Phase 1 and phase 2
NCT03808675	VA Office of Research and Development	Late Parkinson’s disease	Aerobic walking	2019-07-01 to 2024-09-30	57	Phase 2 and phase 3
NCT01257945	University of Colorado, Denver	Parkinson’s disease	Aerobic exercise	2003-04 to 2010-07	163	Phase 2
NCT01439022	Oxford Brookes University	Parkinson’s disease	Exercise programme	2011-09 to 2014-09	105	Phase 2

BDNF controls angiogenesis, cognition, and neuroplasticity, which are necessary for the development of learning and memory functions [134,135]. BDNF is broadly expressed in the central nervous system (CNS), within the hippocampus and cortex. Evidence from multiple research projects shows that both acute and sustained aerobic exercise enhances circulating BDNF levels [136]. Moderate-intensity aerobic exercise has been shown to elicit similar BDNF elevations in old age people, reinforcing the role of aerobic exercise in modulating BDNF production [137].

PE stimulates the expression of fibronectin type III domain-containing protein 5 (FNDC5), a membrane protein in muscles, which is cleaved to release a key myokine, called irisin [138]. Irisin crosses the blood-brain barrier (BBB) and increases BDNF in the hippocampal region of the brain [139]. BDNF is a neurotrophin that plays a crucial role in neurogenesis, synaptic plasticity, learning, and memory. The FNDC5/irisin-BDNF pathway is considered a vital molecular link between PE and improved brain function and neuroprotection [140].

Pre-clinical research has demonstrated that exercise enhances BDNF expression, particularly in the hippocampus [141]. The exercise-induced expression of FNDC5 has been demonstrated in the brain and muscles, indicating a potential role for it. BDNF mRNA and protein levels in the brain and skeletal muscle were enhanced because of the exercise [110]. However, BDNF from muscles was not released into the bloodstream, whereas about 70 to 80 % of BDNF from the brain reaches the blood.

Sleiman *et al.* [142] reported that PE stimulates epigenetic modifications that enhance BDNF gene expression. For instance, β-hydroxybutyrate (DBHB), a metabolite elevated during exercise, inhibits histone deacetylases-2 (HDAC2) and histone deacetylases-3 (HDAC3), leading to the transcriptional activation of BDNF [143]. Another key metabolite, lactate, crosses the BBB and boosts hippocampal BDNF expression, a process dependent on sirtuin (SIRT1) activity and linked to the PGC-1α/FNDC5 pathway [144]. Blocking lactate transport or SIRT1 activity diminishes BDNF expression.

NGF, a protein that is involved in the development, maturation, and proliferation of sympathetic and sensory neurons, affects adult inflammatory hyperalgesia [145]. NGF exerts neuroprotective effects by binding to the CNS's tropomyosin receptor kinase (Trk) A and p75 neurotrophins receptor (p75NTR). Nerve growth factor (NGF) activates the phosphatidylinositol 3-kinase (PI3K)/Akt signalling pathway, thereby supporting neuroplasticity, neuronal survival, and intracellular calcium regulation. Experimental studies have shown that NGF overexpression reduces neuronal apoptosis and improves learning and memory performance [146]. In line with these findings, preclinical evidence indicates that moderate-intensity treadmill exercise modulates PI3K/Akt signalling and increases NGF levels in the hippocampus of aged rats [147].

EGF is a hypoxia-responsive protein that is expressed by various cell types such as endothelial cells, glial cells, MACs and skeletal myofibres [148]. VEGF is a key regulator of angiogenesis and vasculogenesis, increasing cerebral blood flow and vascular development. A growing body of evidence shows that physical activity increases brain-derived VEGF levels, stimulates neurogenesis and angiogenesis, and alleviates cognitive impairments in ischemic conditions by promoting progenitor cell growth and neuronal development [149]. Additionally, exercise-induced lactate accumulation activates the hydroxycarboxylic acid receptor 1 (HCAR1), leading to ERK1/2 and Akt pathway activation and further upregulation of VEGF expression in the hippocampus [150]. In the skeletal muscle-specific VEGF knockout model, PE enhances VEGF levels in the hippocampus, suggesting that muscle-derived VEGF may cross the BBB and promote brain angiogenesis and neurogenesis [112].

A protein called IGF shares structural similarities with insulin. In the adult hippocampus, serum IGF-1 serves as a growth factor that regulates neurogenesis, synaptic plasticity, and neurotransmission [151]. Cognitive decline is linked to age-related decreases in IGF-1 gene expression, whereas cognitive impairment and depressed behaviour are caused by low serum IGF-1 levels. Muscle-derived IGF raises IGF-1 in the hippocampus region and improves mitochondrial function [152]. Serum IGF-1 can penetrate the brain due to the high permeability of IGF-1 across the blood-brain barrier and enhances IGF-1 uptake compared to other neurotrophic factors [153]. PE restores IGF-1 by altering cytokine production and decreasing neurodegeneration.

GDNF is a 134-amino-acid protein that was first discovered for its capacity to ensure the lifespan of dopaminergic and motor neurons in the midbrain [154]. This neurotrophic factor acts by binding to the GDNF family receptor (GFR co-receptors) and by activating receptor tyrosine kinase, PI3K, Erk, and protein kinase B (Akt) signalling. GDNF is considered one of the most potent neurotrophins for maintaining neuromuscular function and enhancing neuroplasticity [154,155]. GDNF expression is upregulated during PE. Pre-clinical studies report an increase in GDNF levels in the spinal cord and muscle of rats by PE [156]. In trained mice, the size of motor neurons and the number of GDNF-containing vesicles both increased, indicating that exercise had a beneficial effect on GDNF expression. Human studies have shown elevated GDNF and NGF levels in muscle biopsies after resistance exercise, accompanied by increased immune cell infiltration [157]. High-intensity exercise is useful in producing neuromuscular junction plasticity and altering the protein composition of GDNF [158].

NT-3 is an amino acid protein with 257 amino acids, which is highly expressed in the period of embryo formation and gradually declines in the postnatal stage [159]. NT-3 is also limited in the dentate gyrus of adult hippocampus and enhances learning and memory through synaptic plasticity. Moreover, NT-3 plays a vital role in the maturation of the neuromuscular junction, synaptic transmission, and survival, as well as the functioning of the sensory neurons. According to pre-clinical research, PE restores NT-3 levels and enhances motor and cognitive behaviour in rats with traumatic brain injury [160]. PE increases NT-4 and Trk-B and promotes their neuroprotective action. According to clinical research, resistance and combined exercise considerably increase NT-3 and NT-4 levels, whereas high-intensity exercise shows no significant effect [161]. Overall, all this evidence emphasises the role of PE in upregulating NT-3 and NT-4, which are key factors in neurogenesis and synaptic plasticity.

#### Epigenetic mechanisms

Emerging research highlights that PE induces epigenetic changes that regulate gene expression and support neuroplasticity [162]. PE influences histone modifications by histone acetylation, which is associated with a more transcriptionally active chromatin environment. Emerging evidence suggests that PE influences epigenetic regulation of the BDNF gene by altering promoter DNA methylation, potentially leading to persistent upregulation of BDNF. These epigenetic changes provide a mechanistic explanation for the long-term neurofunctional benefits observed following exercise exposure, including enhanced brain resilience against neurodegenerative disorders [27].

#### Myokines

Skeletal muscles are recognized as secretory organs that release signalling molecules, including myokines, cytokines, and peptides, which exert autocrine, paracrine, and endocrine effects. Myokines are composed of a group of bioactive molecules released by muscle fibres during contraction during PE. Skeletal muscle constitutes nearly 40 % of human body mass, making it a major contributor to the exercise-induced signalling. Consequently, PE has a substantial influence on the secretion of myokines. Previous research has shown that PE promotes the release of various myokines in muscle tissue, including irisin, PGC-1α, and cathepsin B, thereby facilitating communication between skeletal muscle and the brain [163]. Additionally, fibroblast growth factor 21 (FGF-21), SPARC, and Interleukin-6 (IL-6) are emerging as novel brain-beneficial myokines [164]. These myokines have multiple health benefits associated with regular PE, including improved metabolism, increased muscle mass, reduced inflammation, enhanced neuroplasticity, and better brain health.

PE stimulates the activation of PGC-1α, a central regulatory factor involved in the neuroprotective effects of exercise [165]. Evidence indicates that PGC-1α is abundantly expressed in tissues with high metabolic demand, including neurons, skeletal muscle fibres, and cardiac muscle cells. In the HD models, it mitigates behavioural and sensorimotor deficits by reducing excitotoxicity and extra-synaptic NMDA receptor expression [166]. Furthermore, PGC-1α exerts neuroprotective effects in PD and other neurodegenerative conditions by preserving dendritic spine architecture and maintaining synaptic stability in the hippocampus.

PE induces PGC-1α-dependent expression and cleavage of FNDC5, yielding the circulating hormone irisin. Irisin facilitates the conversion of white adipose tissue into brown-like, thermogenically active fat, a process linked to beneficial effects on the brain. Animal studies demonstrate that direct central exposure to irisin limits neuronal apoptosis, elevates BDNF expression, and improves depression-like behaviours [167]. PE raises plasma irisin levels, penetrates the BBB, and enhances synaptic plasticity, cognition, and neurogenesis [87].

A PGC-1α-regulated myokine, cathepsin B, a lysosomal cysteine protease, is involved in autophagy and lysosomal brain clearance [168]. The level of cathepsin B increases in both brain microglia and serum. PE increases cathepsin B levels in muscle and plasma and improves hippocampus-dependent memory [169]. Cathepsin B can penetrate the blood-brain barrier and increase the expression of doublecortin and BDNF, which are involved in neuronal survival and brain growth, respectively. PE increases Cathepsin B expression in the brain; this effect is enhanced by peripheral cathepsin acting as a myokine [170].

A bone glycoprotein, osteonectin (SPARC), regulates bone mineralisation and mineral crystal formation. SPARC, a myokine involved in tissue repair and tissue regeneration, also plays an important role in collagen production and remodelling [171]. SPARC reduces depressive behaviour and acts synergistically with BDNF to enhance retinal ganglion cell growth via Akt and Erk1/2 phosphorylation. Although it’s unclear whether muscle-derived SPARC crosses the BBB, its exercise-dependent secretion makes SPARC a potential candidate.

PE induces the release of interleukin-6 from skeletal muscle. It has both proinflammatory and anti-inflammatory properties. It is also expressed in the hypothalamus, playing a crucial role in neuro-immune communication. IL-6 is involved in neurogenesis and neuronal regeneration in the brain. IL-6 knockout mice reduced neurogenesis [37]. While overexpression in astroglia surprisingly did not enhance new cell formation, Hyper-IL-6 (a fusion protein of IL-6 and IL-6R) was found to promote neuronal differentiation and glycogenesis via MAPK/CREB signalling. Along with this, it protects against neuronal apoptosis during ischemic brain injury, indicating its critical anti-apoptotic and neuroprotective role [172].

#### Neuroimmune and inflammatory mechanisms

Neuroinflammation plays a central role in the development and progression of neurodegenerative disorders, making the anti-inflammatory effects of exercise essential for the management of the disease. Regular PE enhances neuroimmune communication and promotes neuronal survival. By regulating inflammatory pathways, consistent PE slows disease progression and underscores its value as a therapeutic approach for brain health and neuroprotection.

#### Anti-inflammatory mediation

PE exerts anti-inflammatory effects both peripherally and centrally [173]. While acute exercise may transiently increase pro-inflammatory cytokines, long-term or regular exercise is associated with a reduction in systemic inflammatory markers, including tumour necrosis factor-α (TNF-α), interleukin-6 (IL-6) and C-reactive protein (CRP) [174,175]. In the brain, PE demonstrates neuroprotective properties by decreasing pro-inflammatory cytokine levels in regions such as the hippocampus while promoting anti-inflammatory signalling [176]. Clinical research indicates that chronic systemic inflammation is a common feature of many neurological conditions, such as depression, PD, AD, and HD [177]. Chronic inflammation leads to metabolic and vascular dysfunction, such as endothelial damage, insulin resistance, and neuroinflammation. Chronic brain inflammation can commonly be linked to the aggregation of senescent cells, which release pro-inflammatory cytokines. and are the cause of neuronal dysfunction [178]. Exercise may reduce this inflammatory burden by limiting senescent cell accumulation or modulating senescence-associated secretory phenotypes (SASP), thereby enhancing its neuroprotective effects [179]. Overall, PE is increasingly recognised as a potent non-pharmacological anti-inflammatory intervention. According to some research, PE can directly modulate inflammatory cytokine production and decrease harmful adipokine levels through muscle-adipose cross-talk [180]. In addition, exercise activates the sympathetic nervous system, further contributing to its anti-inflammatory and neuroprotective actions [181]. Animal studies in AD rodent models consistently show that physical exercise, such as treadmill and swimming protocols, mitigates key pathological features of the disease. Exercise improves cognition, enhances hippocampal neurogenesis, and reduces neuroinflammation by lowering the levels of TNF-α, IL-1β, and IL-6. It also restores neurotrophin levels (BDNF, GDNF, NGF, NT-3), decreases neurotoxic tryptophan metabolites, and reduces Aβ deposition and tau phosphorylation [182]. Overall, PE provides robust multidimensional neuroprotection in AD models. Exercise strengthens the HSP70/NF-κB/IL-6/synapsin I axis, preventing inflammation-induced neurological impairments in a traumatic brain injury model [181]. Exercise increases irisin and adiponectin, which play significant roles in suppressing inflammation and promoting brain health, particularly in relation to depression and neurogenesis [183].

Controlled and randomized studies examining exercise-induced inflammatory changes in AD patients are still limited. A two-month aerobic program improved quality of life and reduced systemic TNFα levels. A more recent 16-week RCT with 198 participants showed mixed results: a slight rise in plasma IL-6, reduced IFNγ in APOE ε4 carriers, no significant changes in CSF cytokines, and an unexpected increase in the microglial activation marker TREM2. These findings underscore the need for future studies to refine exercise duration and modality, recruit larger cohorts, and evaluate responses across various stages of AD [1].

#### Antioxidant mediation

PE contributes to neuroprotection by regulating oxidative stress, a key pathological mechanism of many neurodegenerative diseases. Regular PE improves the body’s endogenous antioxidant capacity, thereby mitigating oxidative injury within the neural tissues. In particular, PE increases the activity of antioxidant enzymes, including SOD, CAT, and glutathione, which act to neutralize reactive oxygen species and protect neurons from cellular injury [184]. Along with this, it also influences neuronal homeostasis by modulating both intracellular and extracellular heat shock proteins, notably iHSP70 and Ehsp70 [185]. In neurodegenerative conditions, impaired iHSP70 responses to oxidative stress and protein misfolding weaken cellular resilience. Regular physical activity appears to mitigate this vulnerability by strengthening molecular chaperone systems, with elevated eHSP70 levels providing additional protection, particularly in motor neurons. Together, these antioxidant and stress-response mechanisms reduce neuronal apoptosis, enhance neuroplasticity, and decrease the neurodegenerative process, highlighting exercise as a potential strategy to delay the progression of PD and related disorders.

## Clinical trials

Several clinical trials are currently investigating the neuroprotective benefits of PE. individuals affected by different neurodegenerative conditions to translate promising pre-clinical findings into clinical benefits. These studies employ different exercise approaches, including aerobic, resistance, and combined training, to evaluate their effects on cognition, motor functionality, neuroplasticity, and disease progression. In AD, PD, and other neurodegenerative disorders, published clinical trials especially emphasize the impact of exercise on the brain structure, neurotrophic factor expression and functional outcomes. Some of these trials also explore the underlying cellular and molecular mechanisms responsible for exercise-mediated neuroprotection across the different neurodegenerative disorders, which are summarized in Table 3.

## Conclusion & future perspective

Regular PE demonstrates a positive effect on the general health and well-being of an individual. Aerobic exercise maintains the synaptic plasticity and modulates the development of new neurons, and protects the neural network, causing improved memory, learning and motor functioning. PE helps in the reduction of oxidative stress and neuroinflammation. PE ameliorates and enhances the insulin sensitivity, levels of neutrophins and neurotransmitters. In patients with PD and AD, PE slows the rate of progression and improves motor and cognitive function by reducing protein aggregation and exerting neuroprotective effects.

If the translational point of view is considered, the effective dose, frequency, and duration of exercise should be optimized for an individual to achieve optimal neuroprotective action. This may lead to precision prescription for an individual considering his/her genetics, specific disease status and metabolic condition. Similarly, pharmacological agents with a biosimilar effect to PE need to be designed in the near future to reproduce PE's cellular and molecular effects. Future research paths should be widened to explore the connection between peripheral systems, including muscles, metabolism and immunity, with the central nervous system during exercise. Finally, establishing the connection between PE and clinical practice may help in the integration of PE into evidence-based, comprehensive strategies to manage neurodegenerative diseases.
